# Leptin Reduces the Expression and Increases the Phosphorylation of the Negative Regulators of GLUT4 Traffic TBC1D1 and TBC1D4 in Muscle of *ob/ob* Mice

**DOI:** 10.1371/journal.pone.0029389

**Published:** 2012-01-09

**Authors:** Neira Sáinz, Amaia Rodríguez, Victoria Catalán, Sara Becerril, Beatriz Ramírez, Andoni Lancha, Emma Burgos-Ramos, Javier Gómez-Ambrosi, Gema Frühbeck

**Affiliations:** 1 Metabolic Research Laboratory, Universidad de Navarra, Pamplona, Spain; 2 Department of Endocrinology and Nutrition, Clínica Universidad de Navarra, Pamplona, Spain; 3 Department of Endocrinology, Hospital Infantil Universitario Niño Jesús, Madrid, Spain; 4 CIBER Fisiopatología de la Obesidad y Nutrición (CIBERobn), Instituto de Salud Carlos III, Pamplona, Spain; Universita Magna-Graecia di Catanzaro, Italy

## Abstract

Leptin improves insulin sensitivity in skeletal muscle. Our goal was to determine whether proteins controlling GLUT4 traffic are altered by leptin deficiency and *in vivo* leptin administration in skeletal muscle of wild type and *ob/ob* mice. Leptin-deficient *ob/ob* mice were divided in three groups: control, leptin-treated (1 mg/kg/d) and leptin pair-fed *ob/ob* mice. Microarray analysis revealed that 1,546 and 1,127 genes were regulated by leptin deficiency and leptin treatment, respectively. Among these, we identified 24 genes involved in intracellular vesicle-mediated transport in *ob/ob* mice. TBC1 domain family, member 1 (*Tbc1d1*), a negative regulator of GLUT4 translocation, was up-regulated (*P* = 0.001) in *ob/ob* mice as compared to wild types. Importantly, leptin treatment reduced the transcript levels of *Tbc1d1* (*P*<0.001) and *Tbc1d4* (*P* = 0.004) in the leptin-treated *ob/ob* as compared to pair-fed *ob/ob* animals. In addition, phosphorylation levels of TBC1D1 and TBC1D4 were enhanced in leptin-treated *ob/ob* as compared to control *ob/ob* (*P* = 0.015 and *P* = 0.023, respectively) and pair-fed *ob/ob* (*P* = 0.036 and *P* = 0.034, respectively) mice. Despite similar GLUT4 protein expression in wild type and *ob/ob* groups a different immunolocalization of this protein was evidenced in muscle sections. Leptin treatment increased GLUT4 immunoreactivity in gastrocnemius and extensor digitorum longus sections of leptin-treated *ob/ob* mice. Moreover, GLUT4 protein detected in immunoprecipitates from TBC1D4 was reduced by leptin replacement compared to control *ob/ob* (*P* = 0.013) and pair-fed *ob/ob* (*P* = 0.037) mice. Our findings suggest that leptin enhances the intracellular GLUT4 transport in skeletal muscle of *ob/ob* animals by reducing the expression and activity of the negative regulators of GLUT4 traffic TBC1D1 and TBC1D4.

## Introduction

Skeletal muscle constitutes the tissue with the highest insulin-stimulated glucose uptake. Of several potential facilitative glucose transporters (GLUTs) the GLUT4 isoform is primarily expressed in insulin-responsive tissues, including skeletal muscle and adipose tissue. Insulin stimulates the traffic of GLUT4-containing vesicles to the cell surface and induces their fusion to the plasma membrane [1]. GLUT4 is a continuosly recycling membrane protein with defects in the translocation of GLUT4 from intracellular depots to the plasma membrane being associated with insulin resistance in muscle cells and adipocytes [2].

A key signal transduction pathway for GLUT4 translocation is the Akt-induced phosphorylation of Rab GTPase-activating protein (GAP) AS160, also known as TBC1 domain family, member 4 (TBC1D4) [3]. The phosphorylation of TBC1D4 suppresses its GAP activity, thereby triggering the machinery for the docking of GLUT4 vesicles to the plasma membrane [4]. More recently, several groups have characterized a paralog of TBC1D4 important for regulating body weight and linked to obesity, known as TBC1D1, that is predominantly expressed in skeletal muscle [5], [6]. Both TBC1D4 and TBC1D1 have been defined as negative regulators of GLUT4 vesicle translocation to the plasma membrane [7]. On the other hand, the fusion of GLUT4-containing vesicles with the plasma membrane in muscle and fat cells is mediated by SNAP-associated receptor (SNARE) proteins, such as synaptosomal-associated protein 23 (SNAP23), syntaxin 4, and vesicle-associated membrane protein 2 (VAMP2). This process can be negatively regulated by Munc18c (mammalian homologue of Unc-18) and syntaxin 4-binding protein (Stxbp4, also known as Synip) [8], [9].

Leptin, the product of the *ob* gene, is an adipose-derived hormone that acts as an afferent signal in a negative-feedback loop inhibiting the appetite and regulating adiposity [10], [11]. In addition to its central function as a satiety factor, many peripheral effects of leptin on metabolism have been described [12], [13]. Leptin increases insulin sensitivity in normal and diabetic murine models [14], [15]. The hormone increases the basal and insulin-induced glucose uptake in isolated muscles of rodents as well as in cultured cells in a PI3-kinase (PI3K)-dependent manner [16], [17]. Furthermore, activation of AMP-activated protein kinase (AMPK) stimulates the gene expression of GLUT4 [18] and promotes its translocation to the sarcolemma [19] with leptin activating AMPK in skeletal muscle [20], [21].

In this study, we show for the first time, the *in vivo* participation of leptin in the regulation of gene expression and phosphorylation of TBC1D1 and TBC1D4, key molecules in the traffic of GLUT4 in skeletal muscle of leptin-deficient *ob/ob* mice.

## Methods

### Animals and Treatments

Ten-week-old male obese *ob/ob* mice (Lep^ob^/Lep^ob^, C57BL/6J) (n = 18) and their lean wild type littermate controls (n = 6) (Harlan, Barcelona, Spain), were housed in a room with controlled temperature (22±2°C) and a 12∶12 light-dark cycle. The *ob/ob* mice were divided in control, leptin-treated and pair-fed groups (n = 6 per group). The wild type as well as the control *ob/ob* and pair-fed *ob/ob* groups received vehicle (PBS), while leptin-treated *ob/ob* mice were intraperitoneally administered with leptin (1 mg/kg/d) (Bachem, Bubendorf, Switzerland) twice daily at 08:00 am and 08:00 pm for 28 days. Wild type, control *ob/ob* and leptin-treated *ob/ob* groups were provided with water and food *ad libitum* with a standard rodent chow (2014S Teklad, Harlan), while daily food intake of the pair-fed *ob/ob* group was matched to the amount eaten by the leptin-treated *ob/ob* group the day before to discriminate the inhibitory effect of leptin on appetite. Mice were sacrificed on the 28th day of treatment by CO_2_ inhalation 20 h after the last PBS or leptin administration (in order to avoid picking up effects reflecting an acute response) and after 8 h of fasting. Fasting blood and the gastrocnemius, extensor digitorum longus (EDL) and soleus muscles in fasted-basal conditions were obtained for biochemical and molecular analyses. The gastrocnemius muscle from one hindleg was removed for mRNA and protein extraction. Triacylglycerol (TG) content in gastrocnemius muscle was measured using a commercially available method (Wako, Osaka, Japan). The contralateral hindleg gastrocnemius, as well as the EDL and the soleus muscles were formalin-fixed for immunohistochemical analyses All experimental procedures conformed to the European Guidelines for the Care and Use of Laboratory Animals (directive 86/609). The protocol was approved by the Ethical Committee for Animal Experimentation of the University of Navarra (080/05).

### Blood Analyses

Serum glucose was analyzed using a sensitive-automatic glucose sensor (Ascensia Elite, Bayer, Barcelona, Spain). Serum TG concentrations were spectrophotometrically determined using a commercial kit (Infinity, Thermo Electron, Melbourne, Australia). Free fatty acids (FFA) concentrations were measured using the colorimetric NEFA C kit (Wako), while glycerol was evaluated by enzymatic methods [22]. ELISA kits were used to assess insulin, leptin (Crystal Chem Inc., Chicago, IL, USA) and adiponectin (BioVendor Laboratory Medicine, Inc., Modrice, Czech Republic) concentrations. Insulin resistance was estimated using the HOMA index (fasting insulin [µU/mL]×fasting glucose [mmol/L]/22.5). Insulin sensitivity was determined using the QUICKI index (1/[log(fasting insulin µU/mL)+log(fasting glucose mg/dL)].

### Microarray

Total RNA was extracted from gastrocnemius muscle samples (20–30 mg) of wild type, control *ob/ob* and leptin-treated *ob/ob* mice using TRIzol™ reagent (Invitrogen, Barcelona, Spain) as previously described [21]. Gene expression analyses were conducted using the Agilent Whole Mouse Genome array (G4121B, Agilent Technologies, Santa Clara, CA, USA), containing more than 41,000 mouse transcripts. Briefly, 1 µg of total RNA from each sample was amino-allyl labelled and amplified using the Amino Allyl MessageAmp II aRNA Amplification Kit (Ambion, Austin, TX, USA). Amplified aRNA was fluorescently labelled using Cy3/Cy5 (Amersham Biosciences, Buckinghamshire, UK) and hybridized to Agilent oligomicroarrays. Two hybridizations with fluor reversal (Dye swap) were performed for each sample (n = 5 per group). Microarray slides were scanned (Gene Pix 4100A scanner, Axon Instruments, Union City, CA, USA) and the images quantified with the software GenePiX Pro 6.0. Gene expression data were analyzed using the GeneSpring GX software v. 7.3.1 (Agilent Technologies). Clustering was accomplished with the Gene and Condition Tree algorithms. Gene Ontology groupings (http://babelomics.bioinfo.cipf.es) and the KEGG website (http://www.genome.ad.jp/kegg/pathway) were used in conjunction with GeneSpring (http://www.agilent.com/chem/genespring) to identify pathways and functional groups of genes modified by leptin deficiency and administration. All microarray data reported are described in accordance with MIAME guidelines. More information regarding the microarray experiments can be found at the EMBL-European Bioinformatics Institute (http://www.ebi.ac.uk/aerep/login. ArrayExpress accession number: E-MEXP-1831).

### Real-Time PCR

For first strand cDNA synthesis, constant amounts of total RNA isolated from the gastrocnemius muscle of wild type, control *ob/ob*, pair-fed *ob/ob* and leptin-treated *ob/ob* were reverse-transcribed as previously described [21]. Specific primers and probes to each target gene were designed using the software Primer Express 2.0 (Applied Biosystems, Foster City, CA, USA) (Table 1). The cDNA was amplified using the TaqMan® Universal PCR Master Mix (Applied Biosystems) and the transcript levels for *Tbc1d1*, *Tbc1d4* and *Slc2a4* (*Glut4*) were quantified by Real-Time PCR (7300 Real-Time PCR System, Applied Biosystems). All results were normalized to the levels of *18S* rRNA (Applied Biosystems) and relative quantification was calculated using the ΔΔCt formula.

**Table 1:** Sequences of the primers and Taqman® probes used in the Real-Time PCR analysis.

Gene Name	Gene Symbol	GenBank accesion number	Oligonucleotide sequence (5′-3′)
Solute carrier family 2 (facilitated glucose	*Glut4*	NM_009204	Forward: GTCCTGAGAGCCCCAGATACCT
transporter), member 4 (Slc2a4)			Reverse: TCCAACTTCCGTTTCTCATCCTT
			Probe: FAM-CCTGCCCGAAAGAGTCTAAAGCGCC-TAMRA
TBC1 domain family, member 1	*Tbc1d1*	NM_019636	Forward: GTCCTGACACCAAAAAGATTGCA
			Reverse: TCTCGGCAGATGAATCCAAAGT
			Probe: FAM-TTGCTCTCAGGGCATCAGACATGTGG-TAMRA
TBC1 domain family, member 4	*Tbc1d4*	NM_001081278	Forward: CACTAAATCAGTCGTGCTGGAGAA
			Reverse: CGACAGATAAAGCCAAAGTGATCA
			Probe: FAM-CCTCTTGTTCTCAGGGAATAAAGCAT-TAMRA

### Preparation of Tissue Lysates

Gastrocnemius, EDL and soleus muscles (10–70 mg) were homogenized in ice-cold lysis buffer (10 mmol/L EDTA·2H_2_O, 50 mmol/L NaCl; 1% Triton X-100, 2 mmol/L phenylmethylsulfonyl fluoride) supplemented with a protease inhibitor cocktail (Complete™ Mini-EDTA free, Roche Molecular Biochemicals, Mannheim, Germany) and phosphate inhibitors (50 mmol/L sodium pyrophosphate, 0.1 mol/L NaF, 10 mmol/L Na_3_VO_4_). Lysates were centrifuged (16,000 *g*, 4°C) for 25 min and protein concentrations determined in the supernatants (Bio-Rad Laboratories, Inc., Hercules, CA, USA).

### Immunoprecipitation

TBC1D1 and TBC1D4 proteins were immunoprecipitated from gastrocnemius lysates (250 µg of total protein) using rabbit polyclonal anti-TBC1D1 and TBC1D4 antibodies (Cell Signaling, Inc., Danvers, MA, USA) overnight at 4°C, followed by incubation with protein A-agarose beads (Roche) for 2 h at 4°C. The mixture was centrifuged (16,000 *g*, 4°C) for 15 s and supernatants were removed. Bead-antibody-protein complexes were washed in lysis buffer and proteins were eluted from protein A-agarose beads by boiling in Laemmli buffer.

### Immunoblots

Muscle lysates (30 µg) and immunoprecipitates were run out in 10% SDS-PAGE, subsequently transferred to nitrocellulose membranes (Bio-Rad Laboratories) and blocked in Tris-buffered saline (10 mmol/L Tris-HCl, 150 mmol/L NaCl, pH 8.00) with 0,05% Tween 20 (TBS-T) containing 5% BSA (Sigma, St. Louis, MO, USA) for 2 h at room temperature (RT). Blocked membranes of lysates and immunoprecipitates were probed with the primary antibodies rabbit polyclonal anti-GLUT4 (Abcam, Cambridge, UK) and rabbit polyclonal anti-phospho-Akt-substrate (PAS) (Cell Signaling), in blocking buffer overnight at 4°C. The antigen-antibody complexes were visualized using horseradish peroxidase (HRP)-conjugated anti-rabbit, anti-mouse (Amersham Biosciences) (Abcam) IgG antibodies in TBS-T containing 5% non-fat dry milk for 1 h at RT and the enhanced chemiluminescence ECL Plus detection system (Amersham Biosciences). Membranes of immunoprecipitates probed with PAS and GLUT4 antibodies were stripped and re-probed with the corresponding total antibody rabbit polyclonal anti-TBC1D1 and anti-TBC1D4 (Cell Signaling). The signals from PAS-TBC1D1, PAS-TBC1D4, GLUT4-TBC1D1 and GLUT4-TBC1D4 were related to total TBC1D1 and TBC1D4 in the immunoprecipitate sample. Mouse monoclonal anti-β-actin (Sigma) was used for normalization of GLUT4 density values from total protein lysates of muscle. Bands were analyzed by densitometric analysis using the Gel Doc-Quantity One 4.5.0., Bio-Rad).

### Immunohistochemistry

Sections (5 µm) of formalin-fixed paraffin-embedded muscles (gastrocnemius, EDL and soleus) were dewaxed in xylene and rehydrated in decreasing concentrations of ethanol. Slides were blocked with 5% normal goat serum (Sigma) diluted in TBS (0.05 mol/L Tris-HCl buffer, 0.5 mol/L NaCl, pH 7.36) for 30 min at RT followed by incubation with the rabbit polyclonal anti-GLUT4 antibody (Abcam) in TBS with 5% normal goat serum overnight at 4°C. After washing, the endogen peroxidase activity was quenched using 3% H_2_O_2_ (Sigma) in distilled water for 20 min at RT. Sections were incubated with goat anti-rabbit EnVision-HRP conjugate (Dako Corporation, Hamburg, Germany) for 20 min at RT after subsequent washing. The immunocomplexes were visualized by adding 0.03% 3,3-diaminobenzidine (Dako) as developing system. Negative control slides with omission of the primary antibodies were included. The reaction was stopped and contrasted with Harris' hematoxylin solution (Sigma). Sections were dehydrated and mounted in DePeX (Panreac, Barcelona, Spain).

### Statistical Analyses

All statistical analyses were performed using SPSS 15.0 (SPSS, Chicago, IL, USA). Differences between groups were assessed by one-way ANOVA followed by LSD tests. As previously outlined, Gene Ontology groupings were used to identify pathways significantly affected by leptin deficiency and administration. Statistical comparisons for microarray data to identify differentially expressed genes across different groups were performed using one-way ANOVA and Student's *t*-tests as appropriate. Data are expressed as mean±SEM. Statistical significance was defined as *P*<0.05.

## Results

### Metabolic profile of wild type and *ob/ob* mice

As expected, leptin treatment corrected the obese and diabetic phenotype of *ob/ob* mice (Table 2). Body weight of leptin-treated *ob/ob* animals was reduced by leptin treatment as compared to the control *ob/ob* (*P*<0.0001) and pair-fed *ob/ob* (*P*<0.0001) groups. Leptin administration normalized both glucose (*P* = 0.961) and insulin (*P* = 0.985) concentrations in leptin-treated *ob/ob* mice compared to wild types. Moreover, leptin increased insulin sensitivity in peripheral tissues, as evidenced by the higher QUICKI index in the leptin-treated *ob/ob* mice than in the control *ob/ob* (*P*<0.0001) and pair-fed *ob/ob* (*P* = 0.007) groups, with no statistical differences between wild type and leptin-treated *ob/ob* animals.

**Table 2:** Biochemical profile of wild type and *ob/ob* mice.

	Wild type	Control *ob/ob*	Pair-fed *ob/ob*	Leptin-treated *ob/ob*
**Body weight (g)**	25.8±0.6	51.0±1.2^d^	34.1±1.3^g^	24.4±0.8^g^ **^,^** k
**Glucose (mg/dL)**	184±34	425±27^d^	187±17^g^	182±25^g^
**TG (mg/dL)**	111±10	154±11^b^	135±8	79±10^a^ **^,^** g **^,^** j
**FFA (mmol/L)**	1.27±0.17	1.33±0.17	2.28±0.63^e^	0.77±0.07^i^
**Glycerol (mmol/L)**	48±8	60±6	54±10	11±3^d^ **^,^** g **^,^** j
**Insulin (ng/mL)**	0.45±0.08	10.03±1.32^d^	2.65±0.63^g^	0.47±0.08^g^ **^,^** h
**Adiponectin (µg/mL)**	33.2±2.0	21.6±1.0^c^	35.3±2.0^g^	40.9±2.3^b^ **^,^** g **^,^** h
**HOMA**	5.5±1.5	252.8±34.5^d^	31.5±8.6^g^	5.5±1.4^g^
**QUICKI**	0.318±0.021	0.201±0.002^d^	0.255±0.013^f^	0.311±0.013^g^ **^,^** i
**TG (mg/g)**	1.15±0.09	1.93±0.14^a^	1.20±0.10^g^	1.20±0.17^g^

Data are mean ± SEM (n = 6 per group). Differences between groups were analyzed by one-way ANOVA followed by LSD test.

a *P*<0.05,

b *P*<0.01,

c *P*<0.001 and

d *P*<0.0001 *vs* wild type.

e *P*<0.05,

f *P*<0.01 and

g *P*<0.001 *vs* control *ob/ob*.

h *P*<0.05,

i *P*<0.01,

j *P*<0.001 and

k *P*<0.0001 *vs* pair-fed *ob/ob*. TG: triacylglycerol; FFA: free fatty acids; HOMA: homeostasis model assessment; QUICKI: quantitative insulin sensitivity check index.

### Leptin alters gene expression of key regulators of GLUT4 traffic

Differential gene expression profiles in the gastrocnemius muscle of wild type, control *ob/ob* and leptin-treated *ob/ob* groups were compared by microarray analysis. Leptin repressed 736 genes that were up-regulated in the control *ob/ob* and increased the transcript levels of 846 down-regulated genes. As shown in Table 3, functional analysis of the genes revealed that a number of these are involved in the insulin signaling pathway and glucose metabolism processes.

**Table 3:** Biological processes according to Gene Ontology (GO) altered by leptin deficiency and leptin administration in the gastrocnemius muscle.

Category	wild type *vs ob/ob*	*ob/ob vs* leptin
	*P* value	*P* value
**GO:15031: Protein transport**	0.971	**0.002**
GO:46907: Intracellular transport	0.997	0.062
**GO:6886: Intracellular protein transport**	0.919	**0.033**
GO:16192: Vesicle-mediated transport	0.985	0.344
**GO:5975: Carbohydrate metabolism**	**0.001**	0.129
GO:7264: Small GTPase mediated signal transduction	0.815	0.247
**GO:44262: Cellular carbohydrate metabolism**	**0.005**	0.064
GO:30705: Cytoskeleton-dependent intracellular transport	0.999	0.164
**GO:6092: Main pathways of carbohydrate metabolism**	**0.010**	0.089
**GO:6006: Glucose metabolism**	**0.040**	0.062
GO:51056: Regulation of small GTPase mediated signal transduction	0.944	0.980
GO:48193: Golgi vesicle transport	0.985	0.263
GO:44275: Cellular carbohydrate catabolism	0.169	0.097
GO:16052: Carbohydrate catabolism	0.169	0.097
GO:6007: Glucose catabolism	0.078	0.081
**GO:6096: Glycolysis**	**0.047**	0.232
**GO:51050: Positive regulation of transport**	0.873	**0.041**
GO:45807: Positive regulation of endocytosis	0.778	0.113
**GO:8286: Insulin receptor signaling pathway**	0.173	**0.033**
GO:42593: Glucose homeostasis	0.317	0.424
GO:1678: Cell glucose homeostasis	0.070	0.104

*P* values (FDR adjusted *P* values) reflect the significance of change in prevalence of genes in each category under the leptin deficiency (*ob/ob*) and leptin administration (leptin) conditions in *ob/ob* mice to the expected prevalence of genes in each category. Statistical significant *P* values are highlighted in bold.

To further characterize the actions of leptin on intracellular GLUT4 traffic, we then decided to focus on the set of genes encoding proteins involved in the vesicle-mediated transport process. Table 4 shows that 24 genes encoding proteins involved in the vesicle-mediated transport between the plasma membrane and the intracellular reservoirs were modified by leptin in *ob/ob* mice. Among them, leptin increased gene expression of the glucose transporters *Slc2a3* (*Glut3*) and *Slc2a12* (*Glut12*), while it decreased the mRNA levels of the inhibitors of GLUT4 translocation *Tbc1d1* and *Tbc1d4*, as compared to control *ob/ob* mice. On the contrary, the expression of the genes involved in the fusion to the membrane of GLUT4-containing vesicles, *Vamp2*, *Vamp4*, *Vamp5* and *Vamp8*, *Stxbp4*, *Stx8* and *Sybl1*, was up-regulated by leptin administration in *ob/ob* animals.

**Table 4:** Genes involved in vesicle-mediated transport altered by leptin in the gastrocnemius muscle of *ob/ob* mice.

GenBank Number	Gene Symbol	Gene Name	Fold change		Ratio
			*ob/ob*	leptin	
**Genes down-regulated by leptin**					
NM_019636	***Tbc1d1***	TBC1 domain family, member 1	1.84	0.80	0.44
AK220347	***Tbc1d4***	TBC1 domain family, member 4	1.19	0.64	0.54
NM_016810	***Gosr1***	Golgi SNAP receptor complex member 1	0.94	0.59	0.63
NM_019661	***Ykt6***	YKT6 v-SNARE protein	0.95	0.64	0.67
NM_133704	***Sec22l2***	SEC22 vesicle trafficking protein homolog A	1.42	1.05	0.74
NM_019829	***Stx5a***	Syntaxin 5	0.79	0.59	0.75
NM_019650	***Gosr2***	Golgi SNAP receptor complex member 2	1.09	0.84	0.77
NM_133854	***Snapap***	SNAP-associated protein	0.99	0.81	0.82
NM_172675	***Stx16***	Syntaxin 16	0.86	0.74	0.86
**Genes up-regulated by leptin**					
NM_019972	***Sort1***	Sortilin 1	0.69	1.18	1.71
NM_178934	***Slc2a12***	Solute carrier family 2 (facilitated glucose transporter), member 12	0.67	1.09	1.63
NM_011505	***Stxbp4***	Syntaxin binding protein 4	1.41	2.24	1.59
NM_016794	***Vamp8***	Vesicle-associated membrane protein 8	1.14	1.65	1.45
AF093064	***Stx8***	Syntaxin-like protein 3I35	1.06	1.53	1.44
AK049750	***Sec22l3***	SEC22 vesicle trafficking protein-like 3	1.00	1.41	1.41
AK018344	***Vamp4***	Vesicle-associated membrane protein 4	0.69	0.89	1.29
NM_011401	***Slc2a3***	Solute carrier family 2 (facilitated glucose transporter), member 3	0.99	1.26	1.27
NM_011515	***Sybl1***	Synaptobrevin like 1	1.02	1.27	1.25
NM_016872	***Vamp5***	Vesicle-associated membrane protein 5	0.98	1.21	1.23
NM_009222	***Snap23***	Synaptosomal-associated protein 23	0.95	1.16	1.22
NM_023348	***Snap29***	GS32 protein	0.77	0.94	1.22
NM_009497	***Vamp2***	Vesicle-associated membrane protein 2	1.15	1.34	1.17
NM_009294	***Stx4a***	Syntaxin 4^a^	0.89	1.03	1.16
NM_011342	***Sec22l1***	SEC22 vesicle trafficking protein-like 1	1.02	1.12	1.10

Differential expression of genes is indicated as fold changes with respect to the wild type group presenting only the genes which were significantly different (*P*<0.05) between the leptin-treated and the control *ob/ob* groups. Ratio: fold change value for leptin-treated *vs* the *ob/ob* group.

### Expression of GLUT4 in skeletal muscle of wild type and *ob/ob* mice

**Figure 1:**
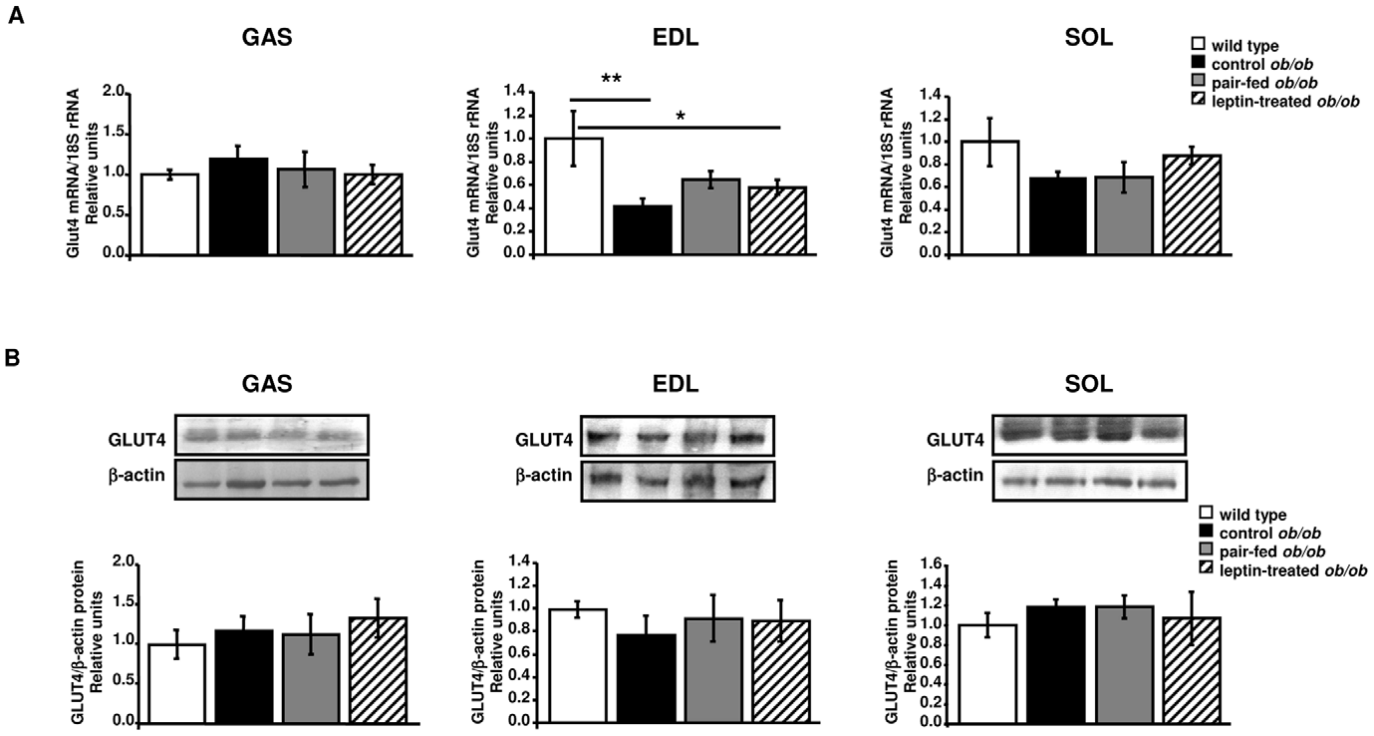
Effect of leptin on glucose transporter GLUT4 expression in skeletal muscle. A: Real-Time PCR analysis of *Glut4* mRNA in the gastrocnemius, extensor digitorum longus (EDL) and soleus muscles of wild type, control *ob/ob*, pair-fed *ob/ob* and leptin-treated *ob/ob* mice (n = 6 per group). B: Representative Western blot analysis of GLUT4 of the gastrocnemius, EDL and soleus muscles of wild type, control *ob/ob*, pair-fed *ob/ob* and leptin-treated *ob/ob* mice. β-actin was used as a loading control (n = 6 per group). Values are presented as means±SEM. * *P*<0.05 by one-way ANOVA followed by LSD tests.

**Figure 2:**
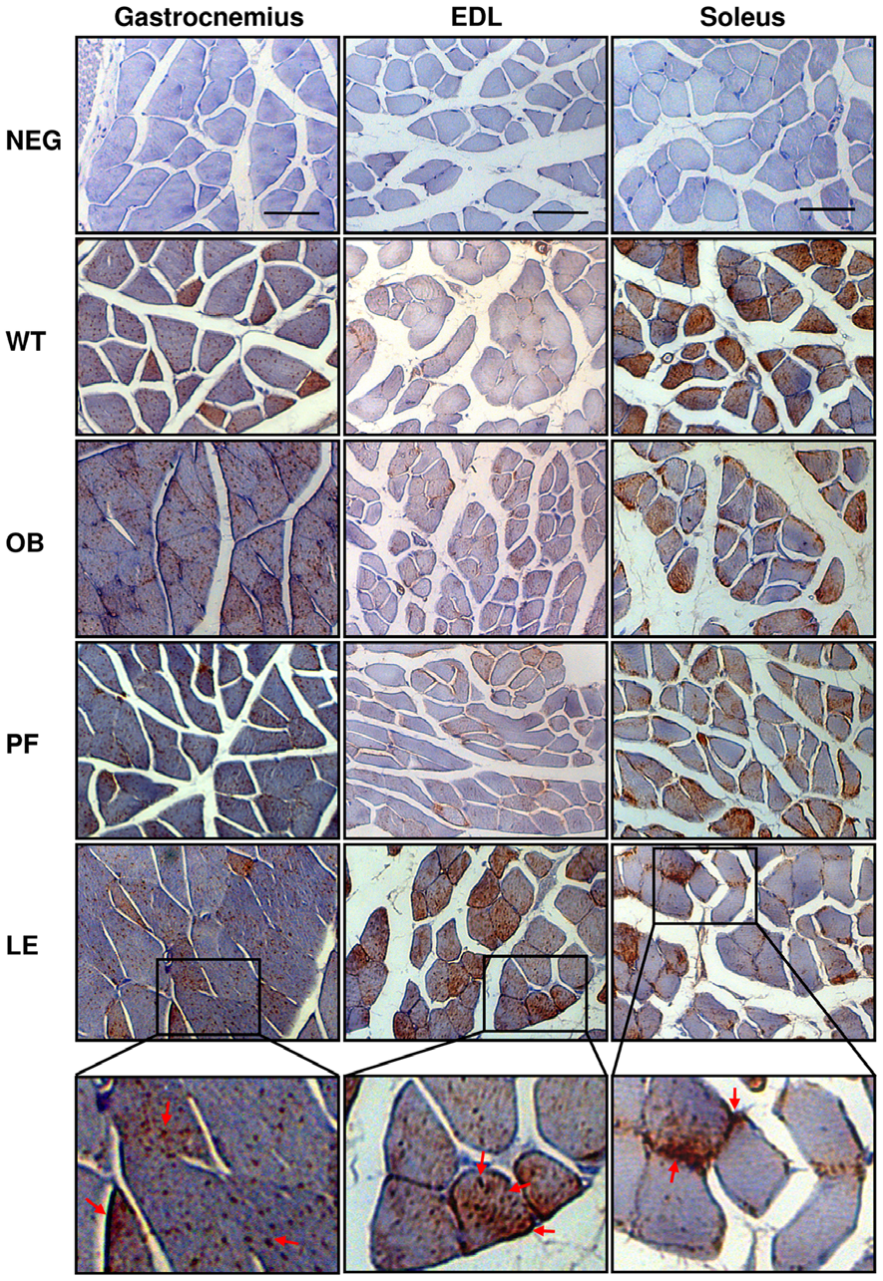
Effect of leptin on the intracellular location of GLUT4 protein in skeletal muscle. Representative images of GLUT4 immunostaining in sections of the gastrocnemius, extensor digitorum longus (EDL) and soleus of wild type (WT), control *ob/ob* (OB), pair-fed *ob/ob* (PF) and leptin-treated *ob/ob* (LE) mice. Larger-magnification images within black frames of immunohistochemical staining for GLUT4 in gastrocnemius, EDL and soleus sections of leptin-treated *ob/ob* mice are shown. Arrows indicate punctuate distribution of GLUT4 vesicles in plasma membrane and cytosol of muscle sections. Scale bars = 50 µm.

No differences for GLUT4 mRNA levels GLUT4 in homogenates of gatrocnemius and soleus muscles between wild type and *ob/ob* groups were found. However, gene expression levels of GLUT4 were reduced in EDL of control *ob/ob* (*P* = 0.007) as compared with wild types, while no effect of leptin administration was detected (Figure 1A). Protein abundance of GLUT4 was not affected by leptin deficiency and leptin administration in gastrocnemius, EDL and soleus muscles of *ob/ob* mice (Figure 1B). To characterize the involvement of leptin on GLUT4 translocation to the plasma membrane an immunohistochemical analysis for the GLUT4 protein in muscle sections of the diverse groups was performed. As shown in Figure 2, a different pattern of GLUT4 immunostaining was detected between muscle sections of wild type and *ob/ob* groups. In wild types, GLUT4 staining was almost exclusively relegated to some gastrocnemius fibers, with typical intracellularly scattered punctate and non-continuous staining observed in the cell surface. In contrast, in leptin-deficient mice (control *ob/ob* and pair-fed *ob/ob* groups) the staining pattern of GLUT4 was uniformly distributed among almost all the gastrocnemius fibers. Interestingly, leptin increased immunoreactivity of GLUT4 in some gastrocnemius fiber sections of the leptin-treated *ob/ob* group as compared to control *ob/ob* and pair-fed *ob/ob* mice, consistent with the GLUT4 staining pattern of wild types. In EDL sections, GLUT4 immunostaining was not altered by leptin deficiency but leptin treatment markedly enhanced immunoreactivity for GLUT4 in muscle sections of leptin-treated *ob/ob* mice. In contrast, a lower immunostaining for GLUT4 was observed in the soleus sections of control *ob/ob* as compared to wild types and no effect of leptin replacement was detected.

### Leptin reduces gene expression levels of *Tbc1d1* and *Tbc1d4* in skeletal muscle of *ob/ob* mice

**Figure 3:**
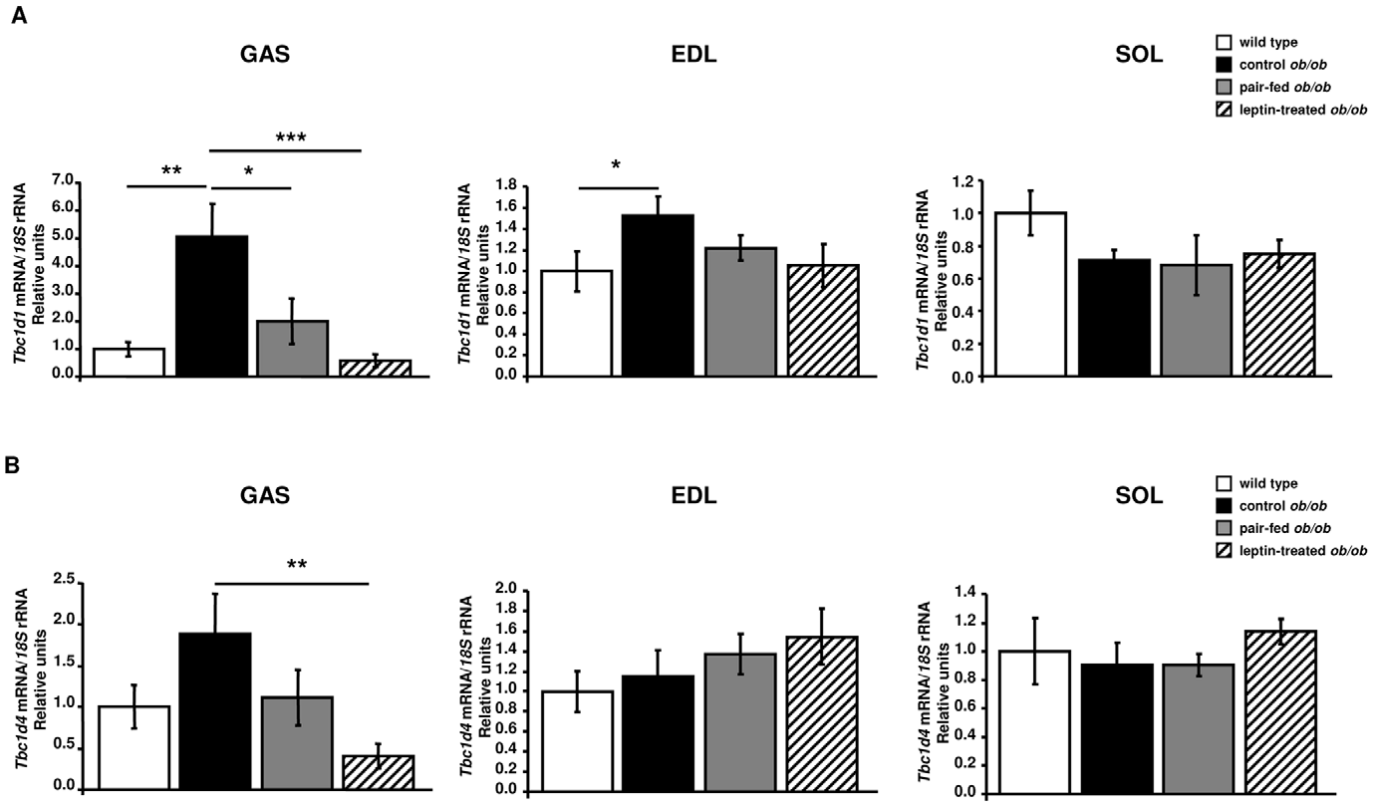
Effect of leptin on the expression in skeletal muscle of the negative regulators of GLUT4 translocation, TBC1D1 and TBC1D4. Real-Time PCR analysis of *Tbc1d1* (A) and *Tbc1d4* (B) mRNA in the gastrocnemius, extensor digitorum longus (EDL) and soleus muscles of wild type, control *ob/ob*, pair-fed *ob/ob* and leptin-treated *ob/ob* mice (n = 6 per group). Values are presented as means±SEM. * *P*<0.05, ** *P*<0.01 and *** *P*<0.001 by one-way ANOVA followed by LSD tests.

Consistently with microarray data, real-time PCR analyses demonstrated that *Tbc1d1* mRNA levels were up-regulated in gastrocnemius of control *ob/ob* mice as compared to wild types (*P* = 0.0012). A trend towards higher transcript levels of *Tbc1d4* between control *ob/ob* and wild type groups was detected. However, no significant differences were observed (*P* = 0.066). Importantly, leptin replacement reduced the expression of *Tbc1d1* (*P* = 0.0005) and *Tbc1d4* (*P* = 0.004) in gastrocnemius of leptin-treated *ob/ob* as compared to control *ob/ob* animals (Figure 3A–B). In addition, higher (*P* = 0.05) transcript levels of *Tbc1d1* were detected in EDL of control *ob/ob* as compared to wild types. No effect of either leptin deficiency or leptin administration was detected for mRNA levels of *Tbc1d1* and *Tbc1d4* in the soleus muscle (Figure 3A–B).

### Leptin increases the phosphorylation of TBC1D1 and TBC1D4 in *ob/ob* mice

**Figure 4:**
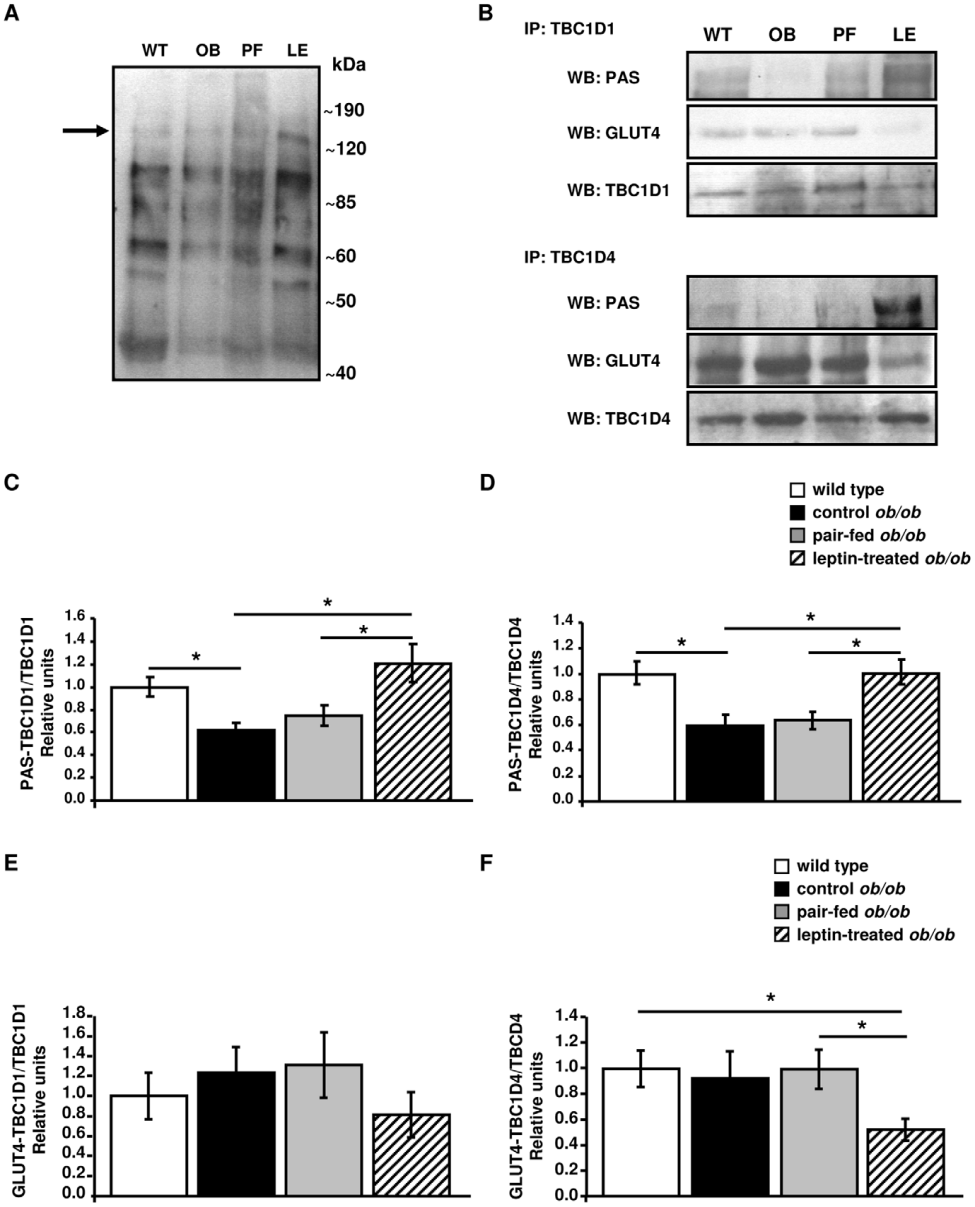
Effect of leptin on the regulation of the negative regulators of GLUT4 translocation, TBC1D1 and TBC1D4. A: Representative phospho-Akt substrate (PAS) immunoblot of gastrocnemius lysate of wild type (WT), control *ob/ob* (OB), pair-fed *ob/ob* (PF) and leptin-treated *ob/ob* (LE) mice. The arrow indicates the band that may correspond to TBC1D1/4. B: Representative blots of immunoprecipitation and immunoblotting experiments. Equal amounts of protein (250 µg) from lysates of the gastrocnemius of WT, OB, PF and LE mice were incubated with TBC1D1 or TBC1D4 antibodies. Immunoprecipitated (IP) samples of TBC1D1 and TBC1D4 were resolved by SDS-PAGE and membranes were immunoblotted (WB) using PAS and GLUT4 antibodies. Membranes of immunoprecipitates probed with PAS and GLUT4 antibodies were stripped and re-probed with the corresponding total antibody TBC1D1 and TBC1D4. The signals from PAS-TBC1D1 and GLUT4-TBC1D1 and PAS-TBC1D4 and GLUT4-TBC1D4 were related to total TBC1D1 and TBC1D4, respectively (n = 6 per group). Leptin increases the phosphorylation levels of TBC1D1 (C) and TBC1D4 (D) in the gastrocnemius of leptin-treated *ob/ob* mice. GLUT4 co-precipitates with intracellular TBC1D1 (E) and TBC1D4 (F) proteins. Values are presented as means±SEM. * *P*<0.05 by one-way ANOVA followed by LSD tests.

The phosphorylation status of TBC1D1 and TBC1D4 in the gastrocnemius of wild type and *ob/ob* mice was determined using the PAS antibody. Figure 4A evidences multiple PAS bands in all the groups and reveals a general increase of the PAS signal in the leptin-treated *ob/ob* group. Among the PAS bands detected in the anti-PAS immunoblot, a band migrating at approximately the 150-kDa location was included. Moreover, this band was reduced in control *ob/ob* as compared to wild type mice and increased after leptin treatment. This band migrated at a location that may correspond to the molecular weight of the TBC1D1 and TBC1D4 proteins. However, the PAS antibody does not distinguish between TBC1D1 and TBC1D4 phosphorylation. Thus, the relative contribution of these two proteins to the PAS signal can not be discerned using this antibody. To resolve this question, protein homogenates of gastrocnemius were immunoprecipitated using TBC1D1 and TBC1D4 antibodies. PAS levels in immunoprecipitate samples of TBC1D1 (Figure 4B and C, *P* = 0.048) and TBC1D4 (Figure 4B and D, *P* = 0.025) were lower in gastrocnemius of control *ob/ob* as compared to wild type animals. No statistically significant differences were observed for PAS-TBC1D1 and PAS-TBC1D4 protein levels between the control *ob/ob* and pair-fed *ob/ob* groups. Importantly, signals from PAS-TBC1D1 and PAS-TBC1D4 were substantially increased after leptin treatment as compared to control *ob/ob* (*P* = 0.015 and *P* = 0.023, respectively) and pair-fed *ob/ob* (*P* = 0.036 and *P* = 0.034, respectively) mice.

### Leptin reduces the immunocomplexes of TBC1D1 and TBC1D4 with GLUT4 in *ob/ob* mice

Since TBC1D1 and TBC1D4 proteins bind GLUT4 to block its translocation from intracellular storage compartments to the plasma membrane, we resolved GLUT4 from the immunoprecipitated samples of TBC1D1 and TBC1D4 in gastrocnemius muscle to examine whether GLUT4 co-precipitates with TBC1D1 and TBC1D4 proteins. As shown in Figure 4B and E-, GLUT4 signals detected in immunoprecipitated samples from TBC1D1 tended to be reduced by leptin treatment as compared to control *ob/ob* and pair-fed *ob/ob* mice, although statistically significant differences were not reached. A similar pattern was observed for GLUT4 in immunoprecipitated samples from TBC1D4, which was reduced in leptin-treated *ob/ob* as compared to control *ob/ob* (*P* = 0.013) and pair-fed *ob/ob* (*P* = 0.037) mice (Figure 4B and F). No statistical differences were detected between control *ob/ob* and pair-fed *ob/ob* groups for GLUT4 proteins in immunoprecipitated samples from TBC1D1 and TBC1D4.

## Discussion

The purpose of our study was to examine the *in vivo* effects of leptin replacement on key molecules regulating GLUT4 translocation in skeletal muscle of the obese and diabetic *ob/ob* mice. Our findings show, for the first time, that leptin administration represses the increased transcript levels and activity of the negative regulators of GLUT4 traffic *Tbc1d1* and *Tbc1d4* in the gastrocnemius muscle of *ob/ob* mice.

Food intake restriction modulates insulin sensitivity and the expression of GLUT4 in human and rodent insulin sensitive tissues [23], [24]. Our data confirm that leptin significantly improves insulin sensitivity in *ob/ob* mice independently of the reduction in food intake. However, the effect of leptin on basal or insulin-stimulated glucose uptake in skeletal muscle has yielded many paradoxical observations [25]. No effect of leptin on glucose uptake was observed in human skeletal muscle cells [26], isolated mouse soleus [27] or rat epitrochlearis muscles [28]. In contrast, several *in vitro* studies have reported that leptin increases basal glucose uptake in C_2_C_12_ muscle cells [16] and in skeletal muscle of mice [29]. Furthermore, *in vivo* leptin treatment increases the glucose uptake in skeletal muscle [17] and improves the insulin sensitivity in normal [30] and diabetic rodents [15] as well as in leptin-deficient *ob/ob* mice [31].

Translocation of GLUT4 from intracellular depots to the plasma membrane of cells is key in glucose uptake, but GLUT4 traffic disruption in human type 2 diabetes has been described [2]. Several studies have examined the role of leptin on GLUT4 translocation showing controversial results. Acute treatment with leptin reduced insulin-stimulated glucose uptake without altering insulin-stimulated GLUT4 translocation in muscle cells of rats [32]. Furthermore, in the absence of insulin, high concentrations of leptin are unable to induce GLUT4 translocation in the soleus of rats [33]. Contrarily, leptin caused a small increase in glucose uptake and movement of GLUT4 to the plasma membrane in C_2_C_12_ muscle cells [16], [34]. In addition, chronic leptin treatment normalized basal glucose transport and GLUT4 content in soleus, but not in the gastrocnemius of high-fat-fed rats [35], as well as the insulin-stimulated GLUT4 content at the cell surface of myotubes without any reduction in total GLUT4 expression [36]. In the present study, GLUT4 mRNA expression was reduced by leptin deficiency in the EDL of control *ob/ob* mice, but it was not modified by leptin administration. In addition, no differences were detected for gene and protein expression levels of GLUT4 between wild type and *ob/ob* groups in gastrocnemius and soleus in spite of the normalization of serum glucose and insulin sensitivity induced by leptin replacement in leptin-treated *ob/ob* animals. A plausible explanation for this finding may relate to the leptin enhancement of GLUT4 activity by inhibiting the activity of TBC1D1 and TBC1D4 and via the alteration of the GLUT4 protein intracellular localization. In this sense, the activity of both TBC1D1 and TBC1D4 can be inhibited by phosphorylation by Akt and AMPK on PAS motif sites in response to insulin or contractile activity [37], [38], [39]. Impaired insulin-induced GLUT4 traffic and phosphorylation of TBC1D4 [40] are restored by endurance exercise-training in skeletal muscle of type 2 diabetes patients [41]. In addition, insulin-stimulated glucose transport and cell surface GLUT4 content are reduced in isolated muscles of mice with *Tbc1d4*-Thr649Ala knockin mutation, but not in adipocytes [42], while inhibition of *Tbc1d4* in 3T3-L1 adipocytes increases the content of GLUT4 in the plasma membrane [43]. In contrast, knockdown of *Tbc1d1* in 3T3-L1 adipocytes does not alter the insulin-stimulated GLUT4 translocation to the plasma membrane, whereas its overexpression partially inhibits the translocation of GLUT4 induced by AMPK [44]. A substantial body of evidence links the activation of AMPK with the reduction of the rate of GLUT4 endocytosis in skeletal muscle [45]. While Akt increases glucose uptake mainly through its action on TBC1D4, AMPK mediates it via the inhibition of TBC1D1 in skeletal muscle of rats [46]. More recently it has been reported that the catalytic AMPK-α2 is the main subunit of AMPK regulating TBC1D1-Ser237 phosphorylation in mice skeletal muscle [47], while disruption of AMPK-α1 signaling prevents the suppressive effects of AMPK activation on TBC1D4 phosphorylation [48]. In this sense, leptin activates AMPK in skeletal muscle [20], [21] and direct or indirect activation of AMPK stimulates the gene expression of GLUT4 [18], [49] and promotes its translocation to the sarcolemma [19]. In this regard, our microarray and real-time PCR data evidenced that administration of leptin suppresses the increased gene expression levels of *Tbc1d1* and *Tbc1d4* in *ob/ob* mice. In addition, leptin replacement enhanced the lower PAS-TBC1D1 and PAS-TBC1D4 protein levels in *ob/ob* mice, at the same time as reducing protein complexes of GLUT4 with TBC1D4. Therefore, given that the phosphorylation of TBC1D1 and TBC1D4 suppresses its GAP activity allowing for the translocation of GLUT4 vesicles to the plasma membrane, our findings indicate that leptin may inhibit the activity of TBC1D1 and TBC1D4 in the gastrocnemius of *ob/ob* mice. Noteworthy, a decrease of the expression of *Tbc1d1* and *Tbc1d4* in gastrocnemius muscle of pair fed *ob/ob*, thought to a lesser extent than in leptin-treated *ob/ob*, was determined. In this respect, given that weight loss has beneficial effects on skeletal muscle insulin sensitivity [23], [24], the possibility that weight loss may alter the TBC1D1 and TBC1D4 expression and/or phosphorylation in skeletal muscle independently of a direct leptin effect should be considered. However, PAS-TBC1D1 and PAS-TBC1D4 levels were increased after leptin administration in leptin-treated *ob/ob* as compared to control *ob/ob* and pair-fed *ob/ob* groups. In addition, several *in vitro* and *in vivo* studies have reported that expression and phosphorylation of TBC1D1 and TBC1D4 in skeletal muscle of rodents [24] and humans [50], [51] are not affected by diet intervention and weight loss. Furthermore, in a previous study, leptin administration did not modify body weight and total TBC1D4 protein expression in soleus of rats, but the hormone was able to recover the *in vitro* impaired insulin-stimulated phosphorylation of TBC1D4 induced by high-fat diet [52]. These studies suggest that modulations of the phosphorylation of TBC1D1 and TBC1D4 do not appear to be early components of the adaptation to weight loss. In this context, discerning whether the improved insulin sensitivity after leptin treatment is associated with an increased insulin-stimulated phosphorylation of TBC1D1 and/or TBC1D4 represents an important topic for future investigations.

In skeletal muscle, TG accumulation leads to insulin resistance. Circulating concentrations of FFA modulate the insulin sensitivity and GLUT4 expression in skeletal muscle with high intramyocellular content leading to insulin resistance [53]. In this sense, serum FFA were not modified by leptin deficiency or administration. In addition, although higher circulating concentrations of FFA and TG were detected in pair-fed *ob/ob* as compared to leptin-treated *ob/ob* mice, no differences for intramyocellular TG content were found. Data suggests an additional effect of leptin on the modulation of the expression and phosphorylation of TBC1D1 and TBC1D4 in *ob/ob* mice independently of the reduction of food intake and circulating concentrations of FFA and TG content in muscle cells.

Consistent with our results, GLUT4 gene and protein expression were not altered in skeletal muscle of insulin-resistant subjects [54] and in skeletal muscle of rats with increased insulin sensitivity after exercise [55], [56], or chronic leptin treatment [17], [57]. Regulation of glucose uptake has been shown to be fiber-type specific in skeletal muscle of rodents [58], [59]. In addition, a muscle-specific fiber-type expression of GLUT proteins has been reported in several studies [60], [61], [62]. To date, 14 members of the GLUT family have been identified, but 3 of them (GLUT4, GLUT5 and GLUT12) account for 98% of the mRNA in human skeletal muscle [62]. Stuart et al. [62] showed that GLUT3, GLUT4 and GLUT12 are predominantly expressed in slow-twitch oxidative fibers of human skeletal muscle. In contrast, the GLUT5 isoform was predominantly detected in fast-twitch glycolytic fibers. Moreover, GLUT12 has been recently identified as a potential candidate for a second insulin-responsive glucose transporter in muscle and fat [63] and a positive regulation of GLUT5 activity and expression by oral administration of leptin has been reported in jejunum of rodents [64]. In the present study, leptin treatment increased transcript levels of GLUT3 and GLUT12 in *ob/ob* animals. These data suggests that GLUT3 and GLUT12 may be key targets for leptin in skeletal muscle of *ob/ob* mice, thereby warranting further studies in this direction.

Paradoxically, leptin increased GLUT4 immunostaining in fiber sections of the glycolytic muscle EDL of leptin-treated *ob/ob* mice, without altering the total GLUT4 protein content by immunoblotting. In addittion, the hormone was unable to modify the GLUT4 staining pattern in the oxidative muscle soleus consistent with immunoblotting results. Controversial results about GLUT4 protein expression by different methods have been detected in several studies [65], [66], [67]. GLUT4 is expressed in different cell types in skeletal muscle tissue. In addition, both fiber-type-dependent and independent factors affect GLUT-4 expression [65], [68] and although EDL is a glycolytic muscle it is composed by different fiber types. Thus, the results on the expression of GLUT4 by immunoblotting and immunostaining in our study would probably be more in accordance performing immunoblotting experiments on single muscle fibers to avoid the influence of another cell type and discriminate the effect of the different fiber types. Furthermore, in the mixed muscle gastrocnemius, total GLUT4 protein content was not different between wild type and *ob/ob* groups, but the GLUT4 immunostaining pattern induced by leptin administration was almost exclusively relegated to some fibers, as compared to leptin-deficient mice (control *ob/ob* and pair-fed *ob/ob* groups), in which the staining pattern of GLUT4 was uniformly distributed among all the gastrocnemius fibers. Leptin appears to increase the GLUT4 signal preferably in some fibers rather than in all of them. Therefore, given that leptin administration was unable to modify the GLUT4 protein abundance in muscle but increased the GLUT4 signal in EDL sections, the lack of differences in GLUT4 protein abundance between groups may be explained by an increase in GLUT4 expression in glycolytic fibers masked by a decrease in the oxidative ones. Another possibility is that leptin alters the intracellular localization of GLUT4 in a fiber-type dependent manner in the gastrocnemius. Accordingly, we can not rule out the possibility that the lack of a leptin effect on GLUT4 mRNA and protein levels may be related to a fiber-type specific effect of the hormone, while warrants future studies for its elucidation.

Subsequently to GLUT4 translocation, a required point for glucose uptake is the fusion of GLUT4-containing vesicles to the plasma membrane. This process is mediated by SNARE proteins [8], [9]. In this sense, the present study shows, for the first time, that leptin administration up-regulates gene expression of proteins involved in the fusion of GLUT4-containing vesicles with the plasma membrane, such as *Vamp2*, *Stx4* and *Stxbp4* in the gastrocnemius of leptin-treated *ob/ob* mice. Leptin also enhanced the transcript levels of *Sort1*, which plays an essential role in the formation of GLUT4 storage vesicles in adipocytes [69] and muscle cells [70]. In addition, overexpression of sortilin in C_2_C_12_ cells enhances the insulin-induced glucose transport by increasing the gene expression levels of GLUT4 [70]. In accordance with our study, a recent report has further shown that mRNA levels of sortilin are down-regulated in adipose tissue and muscles of *ob/ob* and *db/db* mice as well as morbidly obese humans [71].

In summary, our findings provide new insights into the molecular mechanisms triggered by leptin in the regulation of glucose metabolism. Data suggests that leptin replacement improves the defective glucose homeostasis of *ob/ob* mice enhancing the presence of GLUT4 in the plasma membrane of the skeletal muscle cells by reducing the expression and increasing the phosphorylation of the negative regulators of GLUT4 translocation TBC1D1 and TBC1D4.
